# Histone modification pattern evolution after yeast gene duplication

**DOI:** 10.1186/1471-2148-12-111

**Published:** 2012-07-09

**Authors:** Yangyun Zou, Zhixi Su, Wei Huang, Xun Gu

**Affiliations:** 1 Ministry of Education Key Laboratory of Contemporary Anthropology and Center for Evolutionary Biology, School of Life Sciences, Fudan University, Shanghai, 200433, China; 2 Department of Genetics, Development, and Cell Biology, Iowa State University, Ames, IA, 50011, USA

**Keywords:** Histone modification, Histone modification code divergence, Gene duplication, Expression divergence, Epigenetic divergence, *cis*-regulation, *trans*-regulation

## Abstract

**Background:**

Gene duplication and subsequent functional divergence especially expression divergence have been widely considered as main sources for evolutionary innovations. Many studies evidenced that genetic regulatory network evolved rapidly shortly after gene duplication, thus leading to accelerated expression divergence and diversification. However, little is known whether epigenetic factors have mediated the evolution of expression regulation since gene duplication. In this study, we conducted detailed analyses on yeast histone modification (HM), the major epigenetics type in this organism, as well as other available functional genomics data to address this issue.

**Results:**

Duplicate genes, on average, share more common HM-code patterns than random singleton pairs in their promoters and open reading frames (ORF). Though HM-code divergence between duplicates in both promoter and ORF regions increase with their sequence divergence, the HM-code in ORF region evolves slower than that in promoter region, probably owing to the functional constraints imposed on protein sequences. After excluding the confounding effect of sequence divergence (or evolutionary time), we found the evidence supporting the notion that in yeast, the HM-code may co-evolve with *cis*- and *trans*-regulatory factors. Moreover, we observed that deletion of some yeast HM-related enzymes increases the expression divergence between duplicate genes, yet the effect is lower than the case of transcription factor (TF) deletion or environmental stresses.

**Conclusions:**

Our analyses demonstrate that after gene duplication, yeast histone modification profile between duplicates diverged with evolutionary time, similar to genetic regulatory elements. Moreover, we found the evidence of the co-evolution between genetic and epigenetic elements since gene duplication, together contributing to the expression divergence between duplicate genes.

## Background

Although gene duplication has been widely considered as the main source of evolutionary novelties [1-4], the issue of duplicate gene preservation remains a subject of hot debate, i.e., how duplicate copies can escape from being pseudogenized and then evolve from an initial state of complete redundancy to a steady-stable state that both functionally divergent copies are maintained by purifying selection. As one of plausible hypotheses, rapid expression divergence between duplicate genes may be the first step that is fundamental for the preservation of redundant duplicates [3]. There are three recognized types involved in expression regulatory mechanism: (*i*) *cis*-regulation of transcription mediated by promoters, enhancers, silencers, etc.; (*ii*) *trans*-regulation mediated by regulatory proteins binding to *cis* elements, such as transcription factors (TF); and (*iii*) epigenetic regulation, such as DNA methylation, specific histone modification pattern of genes (histone code hypothesis). Many studies have addressed the effect of *cis-* and *trans-* regulatory mechanisms on the expression divergence after gene duplication, e.g., *cis*-regulatory motif, TF-binding interaction, *trans*-acting expression quantitative trait loci (eQTLs) [5-10]. Though there is increasing evidence that epigenetic changes may play important roles in the initial expression divergence between duplicate genes [11-15], little study has been done about how regulatory network between duplicate genes evolve at epigenetic level.

Epigenetic regulation on gene expression is a highly complex process. In a broad sense, it includes DNA methylation, histone modification, nucleosome occupancy, as well as microRNA [11]. Moreover, these epigenetic elements can interact with each other, for instance, the reciprocity between DNA methylation and histone modification [16,17]. The complexity of epigenetic regulation has made it difficult to explore its role in regulatory divergence between duplicates. Nevertheless, we have recognized budding yeast *(Saccharomyces cerevisiae)* as an ideal organism for our purpose, because its epigenetic regulation is relatively simple: DNA cytosine methylation and microRNA were not detected [18,19]. In other words, histone modification is the main representation for epigenetic modification in the budding yeast, less affected by other epigenetic modification types. Therefore, in the present study, we focus on the evolution of histone modification (HM) between yeast duplicate genes.

Eukaryotic DNA with a unit of 146 bp wound around a histone octamer (two copies of each core histone H2A, H2B, H3, H4) is assembled into chromatin. Histone N-terminal tails are subject to multiple covalent post-translational modifications, including lysine (K) acetylation, lysine or arginine (R) methylation, serine (S) phosphorylation, and so on [20,21]. Enormous possible combinations and interactions of histone modification types constitute histone code. The ‘histone code’ hypothesis claims that a specific pattern of hisone modification code can produce a specific effect on local chromatin structure, modulating DNA accessibility, and consequently regulating transcription and other DNA-based biological processes [20-24].

Our goal is to investigate the pattern of yeast histone modification (HM) code divergence between duplicates. Our hypothesis claims (*i*) that, when a gene is duplicated, the gene-specific histone modification profile is also duplicated, so on average duplicate pairs tend to have a higher degree of HM code similarity than randomly selected singleton gene pairs; and (*ii*) that since duplication, HM-code profile between duplicate copies become divergent with evolutionary time. We test these two predictions by conducting genome-wide analyses, as well as genes involved in different biological functions. Moreover, we are particularly interested in whether genetic regulatory elements, including *cis*-motifs (such as TATA box) and transcription factors, and epigenetic HM-code profile co-diverge during the evolution since gene duplication. To this end, time-dependent confounding factors in both genetic and epigenetic factors need to be ruled out. Finally, the significance of our study for having a better understanding of regulatory evolution after gene duplication is discussed.

## Results

Combinational interactions of histone-modifying enzymes (HATs, HDACs, HMTs, HDMs, etc.) to histone N-tail produce numerous types of post-translational modification of histones (H2A, H2B, H3, H4), such as methylation, acetylation [25]. Moreover, the same modification site can be affected by different modifying enzymes, and *vice versa*, generating different histone modification combinations, like H3K4me2 and H3K4ac (dimethylation and acetylation in Lys4 of histone H3, respectively), or H4K8ac (acetylation in Lys8 of histone H4). In this study, histone modification (HM) code of a gene represents the combined profile of different HM sites, HM types and HM states in gene promoter and open reading frame (ORF) regions, respectively (see Methods). We believe that the HM code of a gene reveals the pattern of HM mediated regulatory network of that gene.

### Functional redundancy in histone modification (HM) between yeast duplicate genes

**Figure 1:**
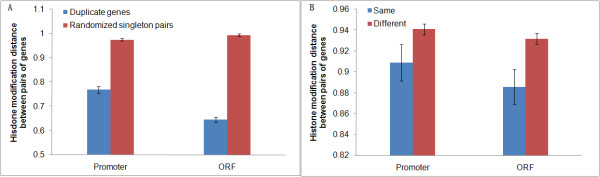
**Comparison of the histone modification divergence between yeast genes.** Comparison of the histone modification divergence between **(A)** duplicate genes and randomized pairs of single-copy genes, and **(B)** all pairs of genes in the same and different chromosomes.

Because of evolutionary relatedness, duplicate pairs may have a higher similarity of histone modification (HM) code than two randomly selected single-copy genes (singletons). To test this hypothesis, we compared the distance of HM code between duplicate pair and randomized singleton pair. To be simple we choose one minus Pearson’s product-moment correlation coefficient, i.e., *D*_*HM*_ = 1-*r*, to define the distance of HM code. The larger the value of *D*_*HM*_, the higher divergence of histone modification code between duplicate genes. Specifically, denote the distance of HM code associated in promoter and ORF regions by *D*_*HM-P*_ and *D*_*HM-O*_, respectively. Randomized pairs were selected from single copy genes with 10000 repeats. As expected, we observed that both *D*_*HM-P*_ and *D*_*HM-O*_ measures show a lower divergence degree of histone modification code between duplicate genes than that of randomized singleton pairs (Wilcoxon rank sum test: *P* < 10^-15^ for both cases; Figure 1A).

Generally speaking, local chromatin environment around genes is one of important components leading to different histone modification code of each gene. While chromatin environment differs in different chromosomes, duplicate genes locating in different chromosomes may be under different chromatin environment, possessing the chromosome-specific HM profile. Following this argument, we classified all yeast gene pairs (duplicate and randomized singleton pairs) under study into two groups: they are located in the same chromosome, or different chromosomes, and tested the relationship between histone modification pattern and chromosome condition. We observed that though the HM profile divergence is not significantly correlated with location distance of gene pairs in the same chromosome (Pearson’s product-moment correlation: *r* = 0.06, *P* = 0.09 for promoter region and *r* = 0.02, *P* = 0.45 for ORF region), gene pairs locating in the same chromosome share more common HM code than that in different chromosomes (Wilcoxon rank sum test: *P* = 0.07 and *P* = 0.01 for promoter and ORF regions, respectively; Figure 1B).

The chromosome effect on the HM-code divergence between duplicate genes may suggest an alternative interpretation about a higher similarity of HM distance between duplicate genes than random pairs. That is, duplicate pairs tend to be located in the same chromosome because of tandem gene duplications, compared to randomized singleton pairs (Chi-squared test: *χ*^2^=17.2, *d.f.* = 1, *P* < 10^-4^). To rule out this possibility, we chose duplicate pairs and randomized singleton pairs where both copies are belonging to different chromosomes, and observed the similar result to Figure 1A (see Additional file 1: Figure S1). Hence, we conclude that duplicate genes represent their functional redundancy at the level of histone modification mediated regulatory network.

### The evolution of HM is coupled with coding sequence divergence after gene duplication

**Figure 2:**
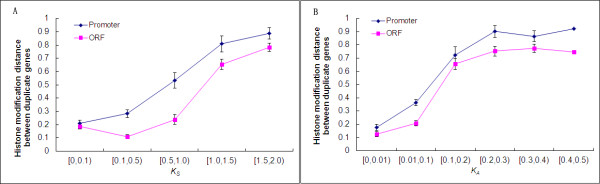
**The divergence of histone modification pattern between duplicate genes (*D***_***HM-P***_**, *D***_***HM-O***_**) increases with synonymous distance *K***_***S ***_**(panel A) or nonsynonymous distance *K***_***A ***_**(panel B) of duplicate genes.**.

We further expect that functional redundancy in histone modification code of duplicate genes as shown in Figure 1A would maintain a high degree in recently duplicated genes, and low in ancient duplicates. To verify this claim, we investigated the relationship between the distance of HM code (*D*_*HM-P*_ or *D*_*HM-O*_) and coding sequence divergence (the synonymous distance *K*_*S*_ or the nonsynonymous distance *K*_*A*_ between duplicate genes). Considering the statistically unreliable estimation of synonymous or nonsynonymous substitution distance when *K*_*S*_ or *K*_*A*_ becomes larger because of repeated substitutions at the same site, we selected duplicate pairs with *K*_*S*_ < 2.0 and *K*_*A*_ < 0.5 for this analysis. We observed that both *D*_*HM-P*_ and *D*_*HM-O*_ are positively correlated with *K*_*S*_ or *K*_*A*_ (Pearson’s product-moment correlation: all *r* > 0.45, *P* < 10^-15^ for all data points; Figure 2), suggesting that the divergence of HM code is coupled with the coding sequence divergence between duplicate genes. To avoid correlated data points bringing the bias, we selected independent pairs of duplicate genes using the method from Zou et al. [9] and reanalyzed. The similar result remains hold (all *r* > 0.40, *P* < 10^-13^ for all data points; in Additional file 1: Figure S2).

Considering *K*_*S*_ or *K*_*A*_ as a proxy to evolutionary time since gene duplication, we suggest that the correlation between the HM-code divergence and the coding sequence divergence has been mainly driven by mutations accumulated with evolutionary time. Our interpretation is based on two reasons: First, we observed a weak negative correlation between the HM divergence and the *K*_*A*_/*K*_*S*_ ratio of duplicate genes (Pearson’s product-moment correlation: *r* = -0.12, *P* < 10^-7^ for *D*_*HM-P*_ and *r* = -0.05, *P* = 0.04 for *D*_*HM-O*_). As the *K*_*A*_/*K*_*S*_ ratio is an indicator of sequence conservation in coding region, our finding implies that duplicate genes with stringent functional constraints on coding sequence may have greater divergence in the HM code, but the effect is marginal. Second, promoter HM code of duplicate genes diverges much quicker than that of ORF HM code (Wilcoxon rank sum test: *P* < 10^-11^; Figure 2), while significant but weak difference of *D*_*HM-P*_ and *D*_*HM-O*_ in randomized singleton pairs (Wilcoxon rank sum test: *P* = 0.02; Figure 1). Some factors may be involved to accelerate the divergence of promoter HM code between duplicate genes, such as the evolution of transcription factors (TF) shared by duplicate genes.

### Co-evolution of the HM-code divergence between duplicates with several genetic regulatory elements

**Figure 3:**
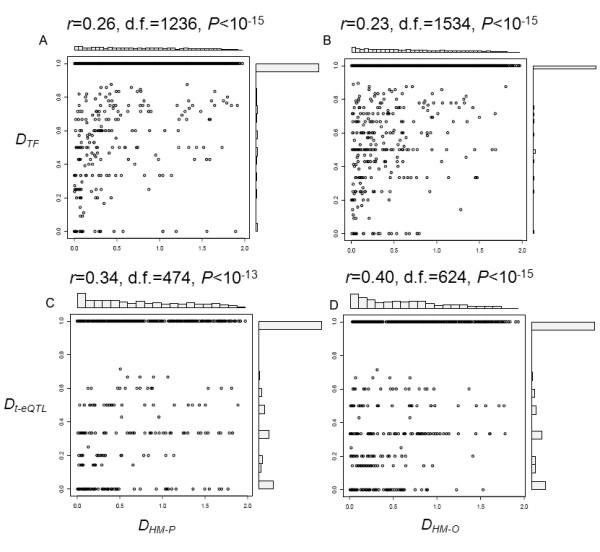
**Relationship between the distance of histone modification pattern and *trans*-regulators shared by duplicate genes. (A)** and **(B)** show the correlation between the distance of transcription factors shared by duplicate genes and the divergence of promoter and ORF histone modification pattern between duplicate genes, respectively, while **(C)** and **(D)** represent the interrelationship between the distance of *trans*-acting eQTLs targeted to the duplicate genes and the distance of promoter and ORF histone modification pattern between duplicate genes, respectively.

The interaction between epigenetic and genetic elements in gene regulation has been increasingly acknowledged [21,26], raising an interesting question whether the divergence of histone modification code between duplicate genes co-evolves with some *trans*-acting factors binding to those duplicate genes. We first studied the relationship between the distance of promoter HM code (*D*_*HM-P*_) and the distance of transcription factors (*D*_*TF*_) and *trans*-acting expression quantitative trait loci (eQTLs) (*D*_*t-eQTL*_) between duplicate genes. *Trans*-acting eQTLs of one gene represent all *trans*-regulatory proteins for its transcription, not limited to transcription factors. Two distance measures *D*_*TF*_ and *D*_*t-eQTL*_ were determined by Czekanowski-Dice formula (Methods). We found that they are significantly correlated (Peasron’s product-moment correlation: *r* > 0.25, *P* < 10^-13^; Figure 3A 3C). Similar results were obtained in the case of ORF HM code (Figure 3B 3D).

As both *D*_*HM-P*_ and *D*_*HM-O*_, as well as *D*_*t-eQTL*_ and *D*_*TF*_, increase with evolutionary time (*K*_*S*_ or *K*_*A*_ as the proxy) [Figure 2; 9], it is reasonable to suspect that *K*_*S*_ or *K*_*A*_ may underlie these statistically significant correlations between the HM code and genetic regulatory elements. We conducted the partial correlation in *D*_*HM-P*_-*D*_*TF*_ and *D*_*HM-P*_-*D*_*t-eQTL*_ of duplicate genes under the controlling of *K*_*S*_ and *K*_*A*_ variables (with the restriction of *K*_*S*_ < 2.0 and *K*_*A*_ < 0.5), and still observed the significant relationship (*r* = 0.18, *P* < 0.001 for *D*_*HM-P*_-*D*_*TF*_ and *r* = 0.15, *P* < 0.05 for *D*_*HM-P*_-*D*_*t-eQTL*_), though they are relatively weak. In short, our analysis provides the evidence that the histone modification code and *trans*-regulators shared by duplicate genes may have co-evolved since gene duplication.

**Figure 4:**
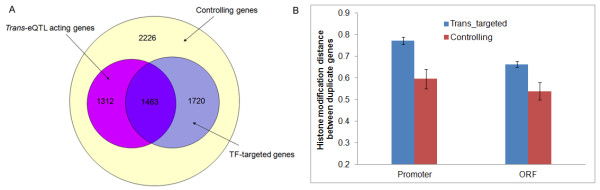
**Different evolutionary rate of the histone modification code for duplicate genes in *trans*-targeted genes and controlling genes. (A)** The information of different category genes, *trans*-targeted genes (TF-targeted genes and *trans*-eQTL acting genes) and controlling genes; **(B)** Comparison of the promoter histone modification distance (*D*_*HM-P*_) and the ORF histone modification distance (*D*_*HM-O*_) of duplicate genes between *trans*-targeted genes and controlling genes. Here, *trans*-targeted genes are the union of TF-targeted genes and *trans*-eQTL acting genes.

Moreover, we design the following analysis to further explore the co-evolution between the HM code and *trans*-regulators (TF or *trans*-acting eQTL), by dividing yeast genes into two categories, *trans*-targeted genes and controlling genes. *Trans*-targeted genes are genes that are targeted by transcription factors (TF-targeted genes) or have at least one *trans*-acting eQTL (*trans*-eQTL acting genes) and the rest of genes are controlling genes (Methods). We totally obtained 4495 *trans*-targeted genes and 2226 controlling genes (Figure 4A). Interestingly, both promoter and ORF HM code distances of duplicates in the group of *trans*-targeted genes are, on average, significantly higher than those in the group of controlling genes (Figure 4B) (Wilcoxon rank sum test: promoter, *P* = 0.003 and ORF, *P* = 0.0001). It should be noticed that the pattern we observed in Figure 4B would not be affected by the strong correlation between the HM divergence and evolutionary time (*K*_*S*_ as the proxy) of duplicate genes, because the distribution of *K*_*S*_ has been found no significant difference between *trans*-targeted genes and controlling genes (Wilcoxon rank sum test: *P* = 0.08).

### The HM-code divergence and TATA-box regulation

**Figure 5:**
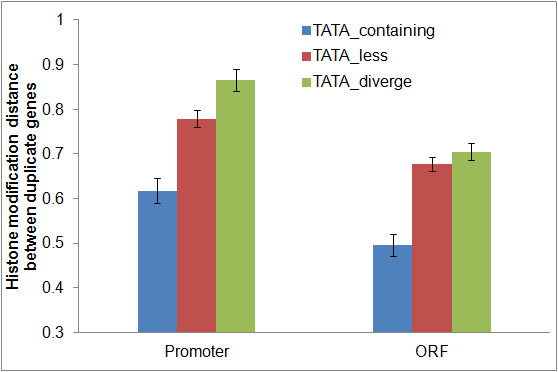
The evolution of promoter or ORF histone modification pattern of duplicate genes which have different status of TATA box.

TATA box is the core promoter element for gene regulation responding to environmental stresses [27,28]. To examine the role of TATA box in the HM-code divergence between duplicate genes, we divided all yeast duplicate pairs into three groups, TATA-containing (both have TATA-box), TATA-less (both do not have TATA-box), and TATA_diverge (only one copy has TATA-box). We compared the HM-code distance in promoter (*D*_*HM-P*_) and ORF (*D*_*HM-O*_) region between duplicate genes in these groups. Interestingly, both *D*_*HM-P*_ and *D*_*HM-O*_ show the highest degree in the TATA-diverge group, and the lowest in the TATA-containing group (Figure 5) (Wilcoxon rank sum test: promoter, *P* < 10^-10^; ORF, *P* < 10^-15^).

Our explanation is as follows. Note that TATA-containing genes may interact with some specific chromatin modification factors to regulate gene expression [29]. In the TATA_diverge group, only one duplicate with TATA-box has such interaction, resulting in a higher HM-code divergence between them. By contrast, in the case of TATA-containing group, both duplicates with TATA-box have similar interactions, resulting in a higher HM-code similarity between them.

### Biological functions and the HM-code divergence between duplicate genes

Do different biological functions affect the level of the HM-code divergence between duplicate genes? We used GO (Gene Ontology) Slim (biological process with 37 categories; see Methods) to address the issue. We analyzed *D*_*HM-P*_ and *D*_*HM-O*_ of duplicate genes in each GO category and compared *D*_*HM-P*_ and *D*_*HM-O*_ among these GO groups by one-way analysis of variance (ANOVA). Our finding is that duplicate pairs with different biological processes differ significantly in *D*_*HM-P*_ and *D*_*HM-O*_ (*P* < 10^-13^ for *D*_*HM-P*_ , *P* < 10^-15^ for *D*_*HM-O*_; Table 1). Use *D*_*HM-P*_ for an example, duplicate genes involved in cofactor metabolic process, cellular amino acid and derivative metabolic process, cell cycle and cytoskeleton organization may have greater HM-code divergence, i.e., a higher *D*_*HM-P*_ , while duplicate genes involved in translation, DNA metabolic process, pseudohyphal growth and transcription may have lower divergence of the promoter HM code. Moreover, we conducted a similar analysis on 24 cellular components (GO Slim classification, see Methods), and observed that duplicate genes in different subcellular localization also undergo the different evolutionary rate of histone modification code after gene duplication (Table 2). It is possible that duplicate genes in some biological processes or subcellular localization are young (measured by small *K*_*S*_) while others are old (with high *K*_*S*_). Since *D*_*HM-P*_ and *D*_*HM-O*_ are positively correlated with *K*_*S*_ (Figure 2), we have to remove the confounding effect caused by *K*_*S*_. We used the analysis of covariance (ANCOVA) (*D*_*HM-P*_ or *D*_*HM-O*_ ~ *K*_*S*_ + T (biological process or subcellular localization) + *K*_*S*_:T) and the result remains statistically highly significant (Table 1, 2), suggesting *K*_*S*_ is not a confounding factor that may affect our analyses.

**Table 1:** The divergence of histone modification code associated with promoter or ORF region of duplicate genes involving in 37 biological processes

**Biological Process**	*** D***_***HM-P***_		*** D***_***HM-O***_	
	** Mean**	** S.E.**^**a**^	** Mean**	** S.E.**
Cofactor metabolic process	1.089857	0.099487	0.94615	0.079585
Cellular amino acid and derivative metabolic process	1.056124	0.075125	0.735075	0.058636
Cell cycle	1.022464	0.070499	0.628888	0.045862
Cytoskeleton organization	1.011731	0.100702	0.732692	0.068568
Carbohydrate metabolic process	0.957513	0.041359	0.801785	0.03478
Cell wall organization	0.943616	0.078784	0.919398	0.069325
Membrane organization	0.943309	0.067702	0.786555	0.069908
Lipid metabolic process	0.93571	0.079815	0.731593	0.059279
Generation of precursor metabolites and energy	0.928247	0.096518	0.882262	0.072135
Anatomical structure morphogenesis	0.921051	0.079036	0.777731	0.073299
Protein modification process	0.900252	0.038873	0.854947	0.030103
Protein folding	0.895056	0.060764	0.677196	0.05632
Response to stress	0.891941	0.051014	0.766131	0.04367
Protein catabolic process	0.877025	0.072138	0.74283	0.065929
Vesicle-mediated transport	0.867052	0.045818	0.763747	0.04406
Cytokinesis	0.855475	0.128721	0.458555	0.075571
Vitamin metabolic process	0.844587	0.123271	0.697339	0.095067
Transport	0.819455	0.026288	0.726211	0.021028
Heterocycle metabolic process	0.812252	0.080271	0.737343	0.070763
Meiosis	0.803064	0.204095	0.586892	0.16534
Cell budding	0.798157	0.088983	0.814543	0.12039
Signal transduction	0.796985	0.069456	0.846847	0.05721
RNA metabolic process	0.792216	0.043033	0.562501	0.032646
Cellular aromatic compound metabolic process	0.783433	0.116406	0.685336	0.092675
Ribosome biogenesis	0.745581	0.078664	0.489313	0.057953
Cellular respiration	0.741278	0.175087	0.970903	0.130576
Conjugation	0.741105	0.115964	0.561448	0.065045
Cellular homeostasis	0.732899	0.107582	0.622864	0.080052
Sporulation resulting in formation of a cellular spore	0.726966	0.119265	0.430464	0.083954
Organelle organization	0.709827	0.043332	0.569849	0.029456
Response to chemical stimulus	0.675718	0.055068	0.590837	0.045851
Nucleus organization	0.668734	0.212327	0.396937	0.134765
Transcription	0.586723	0.061277	0.490756	0.047904
Pseudohyphal growth	0.523636	0.183077	0.741094	0.154572
DNA metabolic process	0.520462	0.057569	0.403555	0.03735
Translation	0.475206	0.058331	0.32502	0.050416
Other	0.803085	0.04149	0.791027	0.036751
ANOVA test	*F* = 3.91, *P* < 10^-13^		*F* = 7.60, *P* < 10^-15^	
ANCOVA test	*F* = 2.24, *P* < 10^-4^		*F* = 4.78, *P* < 10^-15^	

^a^: S.E. denotes the standard error.

**Table 2:** The divergence of histone modification code associated with promoter or ORF region of duplicate genes involving in 24 cellular components

**Cellular Component**	*** D***_***HM-P***_		*** D***_***HM-O***_	
	** Mean**	** S.E.**	** Mean**	** S.E.**
Cytoplasm	0.815386	0.022075	0.691933	0.018111
Mitochondrion	0.804229	0.045602	0.775065	0.037205
Ribosome	0.401566	0.051071	0.21871	0.044488
Nucleus	0.757928	0.03278	0.633467	0.025154
Membrane	0.867299	0.045905	0.719912	0.037068
Mitochondrial envelope	0.850156	0.071138	0.845782	0.058631
Nucleolus	1.041006	0.088386	0.885202	0.076221
Chromosome	0.848459	0.121672	0.500084	0.069019
Membrane fraction	0.728517	0.057793	0.49282	0.052274
Cell wall	0.703015	0.05532	0.397484	0.060315
Vacuole	0.560115	0.090545	0.725808	0.070671
Golgi apparatus	1.020564	0.07273	0.856226	0.060742
Cytoplasmic membrane-bounded vesicle	0.780526	0.149402	0.703323	0.11963
Endomembrane system	0.77575	0.132578	0.712372	0.098021
Cellular bud	0.877352	0.094858	0.65631	0.069712
Cytoskeleton	0.883771	0.094073	0.589399	0.07159
Microtubule organizing center	0.664255	0.193299	0.553038	0.177284
Site of polarized growth	0.875764	0.0889	0.636855	0.064332
Endoplasmic reticulum	0.877175	0.081264	0.673596	0.056244
Plasma membrane	0.72374	0.034621	0.70187	0.027569
Peroxisome	0.500936	0.245856	0.827018	0.120535
Extracellular region	0.553479	0.167985	0.224751	0.129525
Cell cortex	1.00632	0.081755	0.650496	0.076325
Other	0.752484	0.047213	0.632212	0.038696
ANOVA test	*F* = 3.54, *P* < 10^-7^		*F* = 6.53, *P* < 10^-15^	
ANCOVA test	*F* = 1.77, *P* = 0.01		*F* = 4.43, *P* < 10^-10^	

### The expression divergence under genetic, epigenetic and stressful perturbations

**Figure 6:**
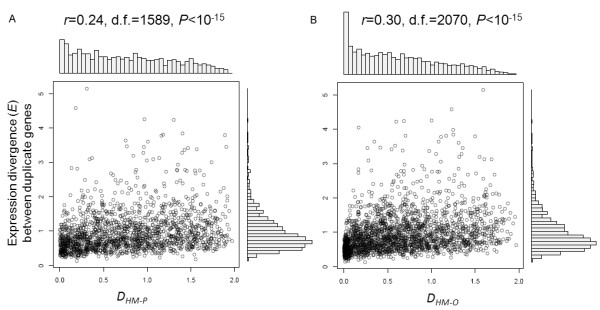
**Correlation between the distance of histone modification pattern and the expression divergence (*E*) of duplicate genes.** Relationship between the expression distance and **(A)** the promoter histone modification divergence (*D*_*HM-P*_) and **(B)** the ORF histone modification divergence (*D*_*HM-O*_).

It is well-documented that gene expression divergence of duplicate genes increases with evolutionary time, but the underlying mechanism remains a subject of debate [30]. The analysis we describe below is to know whether the divergence of HM-mediated regulatory network affects the expression divergence between duplicate genes. We compared the interrelation between the histone modification pattern distance (*D*_*HM-P*_ , *D*_*HM-O*_) and the expression distance (*E*) between duplicate genes, and found that they are significantly correlated (Pearson’s product-moment correlation; *D*_*HM-P*_*E*: *r* = 0.24, *P* < 10^-15^; *D*_*HM-O*_*E*: *r* = 0.30, *P* < 10^-15^; Figure 6).

**Figure 7:**
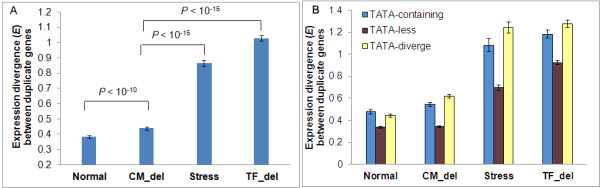
**Comparison of the expression divergence between duplicate genes under different disturbing conditions. (A)** The expression divergence between all duplicate genes; **(B)** The expression divergence between duplicate genes with different status of TATA box. P-value was obtained from Wilcoxon rank sum test using the open-source R statistical analysis language.

We further divided yeast expression profiles into four types from different perturbation conditions: 1) normal developmental or physiological conditions, defined as ‘Normal’ treatment; 2) a set of conditions where expression changes attribute to environmental stresses, denoted as ‘Stress’ treatment; 3) conditions where single gene coding chromatin modifiers (CM) like SWI/SNF, HDACs, HATs, etc. was deleted, denoted as ‘CM_del’ treatment; 4) conditions where single gene coding transcription factors (TF) was deleted, denoted as ‘TF_del’ treatment. The latter two types are able to test the effect of chromatin modification related proteins and transcription factors on other genes, respectively. We then calculated the expression divergence between duplicate genes under these four types of conditions, denoted by *E*_Normal_, *E*_Stress_, *E*_CM_del_, *E*_TF_del_, respectively. We observed that the expression distance between duplicate genes in ‘CM_del’ condition (*E*_CM_del_) is significantly greater than that in ‘Normal’ condition (*E*_Normal_) (Wilcoxon rank sum test: *P* < 10^-10^; Figure 7A), but much lower than that in ‘Stress’ and ‘TF_del’ conditions (*E*_Stress_ and *E*_TF_del_) (Wilcoxon sum rank test, *P* < 10^-15^; Figure 7A). Results imply that histone modification related enzymes and gene associated histone modification profile may indeed influence the expression evolution of duplicate genes, though the relative contribution to the expression divergence between duplicate genes is highly lower than genetic related factors like transcription factors, even externally environmental stresses.

TATA-containing genes are usually enriched in stress-related genes [29], and represent high expression variability [31,32]. In our study, we detected that in four disturbed conditions (Normal, Stress, CM-del, TF_del), the expression divergence in TATA-diverge and TATA-containing groups are significantly larger than that in TATA-less duplicate genes (Figure 7B). The discrepancies between expression change and the histone modification divergence in these duplicate gene types (TATA-containing, TATA-less, TATA-diverge) are observed.

## Discussion

### The divergence of HM profile between duplicate genes

Our detailed analyses on yeast whole-genome histone modification (HM) code profile have shown that duplicate genes share more common HM-code patterns than randomized singleton pairs in their promoter and ORF regions, and the HM-code divergence between duplicates in both regions increase with the sequence divergence. This finding supports the notation that epigenetic divergence between duplicate genes may have been driven by the accumulation of mutations with the duplication time in both their promoter and ORF regions, because it has been shown that the divergence of coding sequence such as *K*_*S*_ between yeast duplicates is a proxy to evolutionary time. In other words, no external driving force is needed to explain the HM profile divergence between duplicates, though it remains possible of neofunctionalization based upon divergent HM profile through some positive selection mechanisms.

### Hypothesis-based genomic correlation analysis

Genome-wide functional analysis of duplicate genes is in attempt to reveal functional correlation between genetic and epigenetic factors during the process of functional innovation through gene duplication. However, the universal confounding effect of the sequence divergence (*K*_*S*_) has complicated the practical analyses. Consequently, the controversy about the cause-effect interpretation has been inevitable, because genome-wide analysis of duplicate genes has been viewed as an exploration rather than a hypothesis-testing approach.

Since the correlation between the HM profile divergence and the sequence divergence actually reflects the fundamental evolutionary process driven by the accumulation of mutations, we view this as a null hypothesis in the genome-wide functional analysis of duplicate genes. That is, any meaningful inference about the functional correlation within or between genetic and epigenetic elements needs to reject this null hypothesis, as we have shown in this study.

### Interaction between genetic and epigenetic elements: who is the driver?

The interaction between epigenetic and genetic elements in gene regulation has been increasingly acknowledged. For instance, the establishment of the histone modification code may partially involve the recruitment of specific histone modifying enzymes such as HATs, HDACs, HMTs by transcription factors (TF) [26]. Meanwhile, the distinctly combinatory histone modification code associated with gene may also provide specific binding code that is read by other transcription factors [21]. These observations raise an interesting question whether the divergence of histone modification code between duplicate genes may co-evolve with *trans*-acting factors binding to those duplicate genes, e.g., transcription factors, histone modifying enzymes.

We have observed a higher divergence for HM code of duplicate genes in the category of *trans*-targeted genes than that of controlling genes, suggesting that the change of *trans*-regulators binding to duplicate genes may affect the pattern of HM code in both promoter and ORF regions, and thus accelerating the divergence of histone modification code between duplicate genes. Yet, it remains unclear about the cause-effect relationship. For instance, does the divergence of *trans*-regulators to duplicate genes facilitate the divergence of histone modification between duplicate genes, or *vice versa*? Our further study will address this issue.

### Functional preference in the HM code divergence between duplicate genes

We observed the functional bias on the HM-code divergence after gene duplication. One possibility is that histone proteins associated with genes in different biological functions may be subject to differentially post-translational modification, leading to different divergence rate of histone modification code between duplicate genes. Since histone modification process of one gene largely depends on its chromatin environment and DNA sequence interacted by histone modifying enzymes [33], functionally selective constraints may also be imposed on histone modification evolution associated with that gene, a situation similar to DNA sequence evolution.

## Conclusions

In this study, we unveiled the evolution of yeast histone modification code since gene duplication. Though duplicate genes represent functional redundancy at histone modification level compared with single-copy genes, the histone modification divergence occurred along with evolutionary time (*K*_*S*_ as the proxy), which possibly due to the coding sequence evolution after gene duplication. Moreover, the histone modification code in ORF region evolves slower than that in promoter region, indicative of functionally selective constraints on protein sequences. Going further, after controlling the confounding effect of the coding sequence divergence (*K*_*S*_), the histone modification code co-evolves with *cis*- (TATA box) and *trans*- (TF and *trans*-acting eQTL) regulatory factors, confirmed by the fact that the histone modification code is shaped by the combined interaction among histone-modifying enzymes, *trans*-acting elements and *cis*-regulatory motif. In addition, histone modification makes contribution to the expression divergence between duplicate genes, despite the minor effect compared to transcription factors and environmental stresses. Taking together, we provided the evidence of the co-evolution between genetic and epigenetic elements since gene duplication, together contributing to the expression divergence between duplicate genes.

## Methods

### Data of yeast histone modification pattern

Genome-wide histone modification pattern data of *Saccharomyces cerevisiae* were downloaded from ChromatinDB [34] ( http://www.bioinformatics2.wsu.edu/cgi-bin/ChromatinDB/cgi/visualize_select.pl). ChromatinDB provides the user with easy access to ChIP-microarray data for a large set of histones or histone modifications in *S. cerevisiae*, which includes 17 distinct histone modification combinations like dimethylation in Lys4 of histone H3 (H3K4me2), acetylation in Lys12 of histone H4 (H4K12ac), etc. and 5 histone protein occupancy levels (H2A, H2B, H3, H4, H2A.Z). We applied log base-2 of average enrichment ratio with nucleosome-normalizing for each of 22 histone modification data in promoter and open reading frame (ORF) regions of genes.

### Yeast microarray expression data

A total of 84 microarray expression data points of *S. cerevisiae* whose expression changes are attributing to internal disturbing like developmental or physiological conditions were respectively collected [35-37]. This type was defined as “Normal” conditions, which are not genetically perturbed by regulatory network related elements like transcription factors and chromatin modifiers (CM) or other environmental stresses. We collected 170 gene expression profiles of yeast strains mutated for various chromatin modifiers from 26 publications [38]. We called this type of expression profile data as ‘CM_del’ conditions. Expression profile data of 263 transcription factor-deletion experiments were obtained from the Gene Expression Omnibus (GEO) database under the series accession number GSE4654 [39]. Similarly, this type was denoted as ‘TF_del’ experiments. A total of 504 cDNA microarray data points of yeast whose expression changes are attributing to environmental stresses were collected [9]. We call this type as ‘Stress’ conditions. Normalization was done as each original paper recommended.

### Data of *trans*- and *cis*- regulatory elements

Transcription factor (TF)-DNA binding profiles of yeast were downloaded from Lee et al. [40] and Harbison et al. [41]. In the study of Harbison et al. (2004), we just used 203 DNA-binding transcription factors in rich media conditions, regardless of 84 regulators in environmental stressed conditions. Most transcription factors in two studies are overlapped. Finally, we obtained 207 TF-all *S. cerevisiae* genes binding interaction profiles. For each gene, a p-value was assigned to measure the probability of true TF-target interaction; a smaller p-value means the interaction is more likely. Here, we used relatively stringent significance level of 0.001 as cutoff to define the status of TF-target gene interaction. Two studies together determine all TF-target gene interactions, and we observed that 3183 genes are binding by at least one transcription factor (TF-targeted genes). Yeast genomic expression quantitative trait loci (eQTLs) data were downloaded from Brem and Kruglyak [42]. Wilcoxon-Mann-Whitney (WMW) test with the criterion of 50 kb interval was conducted to detect and define eQTL regions [9]. Finally, we obtained 2775 genes which at least have one *trans*-acting eQTL (*trans*-eQTL acting genes). Thus, we can divide all yeast genes into two categories, *trans*-targeted genes and controlling genes, where *trans*-targeted genes are the union of TF-targeted genes and *trans*-eQTL acting genes, while controlling genes are the reminders. Since *trans*-eQTL acting genes were not only regulated by transcription factors, but most by chromatin related enzymes and other factors [43], *trans*-targeted genes may have the possibility to be regulated by all *trans*-regulators, not restricted to transcription factors.

There are two types of genes, TATA-containing genes and TATA-less genes [29]. We classified all duplicate pairs into three categories, TATA-containing, TATA-less and TATA-diverge pairs. TATA-containing and TATA-less types are those duplicate pairs where both copies have or don’t have TATA box, respectively, while TATA-diverge type refers to duplicate pairs where one copy belongs to TATA-containing genes, and the other TATA-less genes.

### Protein subcellular localization and biological process

The information of protein localization and biological process for *S. cerevisiae* was defined by the Gene Ontology (GO) classification, and downloaded from Saccharomyces Genome Database. GO Slim was used to classify 24 cellular component sorts and 37 biological process categories. A duplicate gene pair was assigned to a GO Slim term if both duplicate copies are belonging to this GO term, or one copy is belonging to while the other is not annotated.

### Defining functional divergence between yeast duplicate genes

There are two types of histone modification profiles, histone modification pattern associated with gene promoter region and open reading frame (ORF) region. One minus Pearson’s product-moment correlation coefficient (1-*r*) was used to determine the distance of these two types of histone modification pattern between duplicate genes, shortly denoted as *D*_*HM-P*_ for promoter histone modification distance and *D*_*HM-O*_ for ORF histone modification distance.

We modified the Czekanowski-Dice formula [44] to calculate the distance of transcription factors or *trans*-acting eQTLs shared by duplicate gene 1 and 2 in a duplicate pair, shortly denoted as *D*_*TF*_ and *D*_*t-eQTL*_, respectively. Suppose Δ_*12*_ be the number of TFs or *trans*-acting eQTLs that differ between one duplicate pair; *y*_*1*_∪*y*_*2*_ be the number of TFs or *trans*-acting eQTLs that regulate at least one of duplicate genes, and *y*_*1*_∩*y*_*2*_ be the number of shared TFs or *trans*-acting eQTLs between a duplicate pair. Then, the TF distance or *trans*-acting eQTL distance between duplicate genes 1 and 2 is defined as follows:

(1) $$D_{\mathrm{TF}}\left(1,2\right)\;\text{or}\;D_{t-eQTL}\left(1,2\right)=\Delta_{12}/\left[y_{1}\cup y_{2}+y_{1}\cap y_{2}\right]$$

Apparently, the greater the value, the higher degree of TF or *trans*-acting eQTL divergence between duplicate genes.

We used evolutionary distance (*E*) defined by Gu et al. [5] as the measure of expression divergence between duplicate genes of four types, shortly denoted as *E*_Normal_*E*_CM_del_*E*_TF_del_ and *E*_Stress_ for ‘Normal’, ‘CM_del’, ‘TF_del’ and ‘Stress’ experiments, respectively. Specifically, for any duplicate gene 1 and 2, let *x*_1*k*_ and *x*_2*k*_ be its expression level, respectively, in the *k*th microarray experiment; ${\bar{x}}_{1}$and ${\bar{x}}_{2}$be the mean of expression level in *k*th microarray experiments, respectively, where *k = 1,…m.* The formula of the expression distance (*E*) between gene 1 and 2 is as follows:

(2) $${\hat{E}}_{12}=\sum_{k=1}^{m}{\left[\left(x_{1k}-{\bar{x}}_{1})-(x_{2k}-{\bar{x}}_{2}\right)\right]}^{2}/(m-1)$$

### Determination of yeast duplicate pairs

The method of Gu et al. [45] was applied to identify duplicate genes. As the criterion of 80% alignable regions between protein sequences is too stringent, and may miss some duplicate genes, we reduced this criterion to 50%. All pairs of duplicate genes in each gene family were used for the analysis. The reminders of *S. cerevisiae* genes were considered as singleton genes. The rate of synonymous substitutions (*K*_*S*_) and nonsynonymous substitutions (*K*_*A*_) between duplicate genes were estimated using PAML [46] with default parameters.

## Abbreviations

HATs: Histone acetyltransferases; HDACs: Histone deacetylases; HMTs: Histone methyltransferases; HDMs: Histone demethylases; K_S_: The rate of synonymous substitutions; K_A_: The rate of nonsynonymous substitutions; TF: Transcription factor; eQTLs: Expression quantitative trait loci; ORF: Open reading frame; HM: Hitone modification; ANOVA: Analysis of variance; ANCOVA: Analysis of covariance; GO: Gene ontology; CM: Chromatin modifiers.

## Competing interests

The authors declare that they have no competing interests.

## Authors’ contributions

YZ designed and performed the study, analyzed the data and drafted the manuscript. ZS and WH analyzed the data. XG designed the study and drafted the manuscript. All authors read and approved the final version of the manuscript.

## Supplementary Material

Additional file 1 **Figures S1-S2.** are available at online web site of *BMC Evolutionary Biology * journal. Click here for file
