# Supplementary material for: Histone modification pattern evolution after yeast gene duplication
Source: BMC Evol Biol. 2012 Jul 9;12:111. doi: 10.1186/1471-2148-12-111 (PMC3495647; doi:10.1186/1471-2148-12-111)

**Supplementary materials**

**Figure S1** Comparison of the histone modification divergence between duplicate genes and randomized singleton pairs which are belonging to different chromosomes.

**Figure S2** The divergence of histone modification pattern between independent duplicate pairs (*D_HM-P_*, *D_HM-O_*) increases with synonymous distance *K_S_* (panel A) or nonsynonymous distance *K_A_* (panel B) between duplicate genes.

**Figure S1**


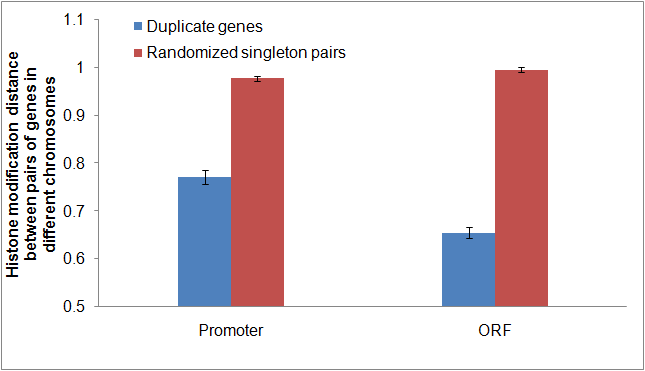


**Figure S2**


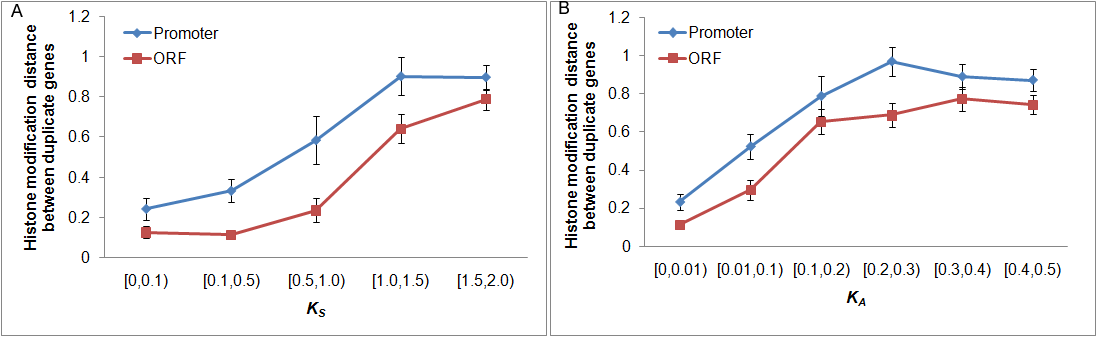

Supplement: Additional file 1 — Figures S1-S2. are available at online web site of BMC Evolutionary Biology journal. [file 1471-2148-12-111-S1.docx]
